# Genomic characterization of bacteria from the ultra-oligotrophic Madison aquifer: insight into the archetypical LuxI/LuxR and identification of novel LuxR solos

**DOI:** 10.1186/s13104-021-05589-6

**Published:** 2021-05-08

**Authors:** Peter C. Wengert, Narayan H. Wong, Hazel A. Barton, Han Ming Gan, André O. Hudson, Michael A. Savka

**Affiliations:** 1 Thomas H. Gosnell School of Life Sciences, College of Science, Rochester Institute of Technology, Rochester, NY 14623 USA; 2 Department of Biology, University of Akron, Akron, OH USA; 3 Centre for Integrative Ecology, School of Life and Environmental Sciences, Deakin University, Geelong, Vic 3220 Australia; 4 GeneSEQ Sdn Bhd, Bandar Bukit Beruntung, 48300 Rawang, Selangor Malaysia

**Keywords:** Madison aquifer bacteria, Karst aquifer, Ultra-oligotrophic bacteria, Pristine cave, Wind Cave National Park, *LuxI*, *LuxR*, *LuxR* solo

## Abstract

**Objectives:**

To characterize the bacterial community of Wind Cave’s Madison aquifer through whole-genome sequencing, and to better understand the bacterial ecology by identifying genes involved in acyl-homoserine lactone (AHL) based quorum-sensing (QS) systems.

**Results:**

Genome-based taxonomic classification revealed the microbial richness present in the pristine Madison aquifer. The strains were found to span eleven genera and fourteen species, of which eight had uncertain taxonomic classifications. The genomes of strains SD129 and SD340 were found to contain the archetypical AHL QS system composed of two genes, *luxI* and *luxR*. Surprisingly, the genomes of strains SD115, SD129, SD274 and SD316 were found to contain one to three *luxR* orphans (solos). Strain SD129, besides possessing an archetypical AHL QS *luxI-luxR* pair, also contained two *luxR* solos, while strain SD316 contained three LuxR solos and no *luxI-luxR* pairs. The ligand-binding domain of two LuxR solos, one each from strains SD129 and SD316, were found to contain novel substitutions not previously reported, thus may represent two LuxR orphans that detection and response to unknown self-produced signal(s), or to signal(s) produced by other organisms.

**Supplementary Information:**

The online version contains supplementary material available at 10.1186/s13104-021-05589-6.

## Introduction

Due to difficulties in access, the microbial life in subsurface aquifers are an under-explored area of microbiology [1]. A recent study has demonstrated that the Madison aquifer, accessed directly by travel through Wind Cave, Wind Cave National Park (WCNP), had a greater bacterial diversity compared to nearby wells that intersected the same aquifer [2]. This discrepancy was shown to be due to contamination of the well water by bacterial species from overlaying rock units, meaning that the microbiology of the aquifer itself could only be accurately assessed via the cave. Without the influence of the well-water microbiology, it was found that the microbiology of the ultra-oligotrophic Madison aquifer was more complex than previously anticipated [2].

Quorum sensing (QS) is a bacterial cell–cell signaling system that employs small compound signals and regulates group behaviors for bacterial-bacterial and bacterial-host interactions [3, 4]. In one QS system, bacteria produce and secrete signals, called acyl-homoserine lactones (AHLs), into the surrounding environment. A typical AHL-QS system contains a LuxI (the AHL signal synthase) and a LuxR (transcriptional regulator). These proteins are usually encoded adjacent to each other on the chromosome [5]. In addition to the canonical *luxI/luxR* pair, many bacteria also contain extra copies of *luxR* transcriptional regulators that are not proximal to any *luxI* synthase gene [5].

An unpaired *luxR* gene is termed a *luxR* solos/orphan and similarly encodes for QS LuxR-type transcriptional regulators consisting of a signal (ligand)-binding domain at the N terminus and a DNA-binding helix-turn-helix (HTH) domain at the C terminus [6–8]. Some solos respond to endogenously produced AHLs to expand their regulatory range. Others “eavesdrop” on other bacterial species, changing their gene expression in response to the foreign AHL signals. There are even examples of LuxR solos responding to other chemical signals entirely, including those produced by species in other kingdoms of life. Such a subfamily of LuxR solos has been identified in plant-associated bacteria (PAB), which respond to plant-produced signals, thus forming an interkingdom signaling circuits [9].

We recently described the whole-genome sequences (wgs) of eight *Ensifer* sp. isolated from two different caves including strain SD006, from the Madison aquifer of WCNP [1]. The genome of SD006 is 427,000 bp larger than the largest of the other seven *Ensifer* sp. isolated from a dry limestone surface of the Lechuguilla Cave in New Mexico [10]. We are not aware of other studies that report bacterial wgs obtained from a subterranean aquifer accessed by a cave with insights on AHL quorum sensing.

In this work, first we provide wgs, de novo genome assembly and annotation of fourteen diverse bacterial strains isolated from the Madison aquifer accessed via Wind Cave [2]. Second, we provide insight utilizing these wgs with various genome-mining and proteomic tools to resolve the questions of strain classification and identity of quorum-sensing genes of the AHL class, *luxI* and *luxR* homologs, using a systematic bioinformatic approach [5, 9, 10]; and finally, we identified seven new LuxR solos from four WCNP strains, SD115, SD129, SD274 and SD316.

## Main text

### Materials and methods

SD strains were isolated from calcite lake in Wind Cave, which represents the piezometric surface of the Madison aquifer where it is intersected by the cave at a depth of 200 m below the surface [2]. The strains were maintained on half-strength tryptic soy agar medium (Merck, Germany).

Genomic DNA was isolated from 2.0 ml of two-day-old broth cultures using Sigma-Aldrich DNA extraction kit according to the manufacturer’s recommendations. Then, 1 ng of DNA from each isolate as quantified using PicoGreen (ThermoFisher Scientific) was processed using the Nextera XT library prep kit (Illumina) followed by sequencing on the Illumina MiSeq (2 × 250 paired-end run configuration).

Adapter-trimmed paired-end reads were assembled de novo using Unicycler tool [11]. The whole genome assemblies were then uploaded to the antibiotics and secondary metabolite analysis shell (antiSMASH) [12], in order to predict and identify secondary metabolite biosynthetic pathways. The assemblies were also uploaded to JSpeciesWS for identification via Tetra correlation search in conjunction with ANIb [13]. Other genes of interest were searched for using tblastn multiple alignment, using reference proteins as query sequences [14]. Phylogenomic analysis was carried-out using PhyloPhlAn [15].

To test for quorum sensing cell–cell communication mechanism of the acyl-homoserine lactone (AHL) class, antiSMASH analysis [11] was performed on each of the SD series genomes to identify secondary metabolites which include *luxI* homologs that encode for the production of AHLs. Tblastn multiple sequence alignment was used to test each genome for the presence LuxR homologies, using query sequence AFP89744.1. Alignments with a MaxScore of 50 or greater were considered putative LuxR homologs.

Putative LuxI and LuxR homologs were first identified based on the presence of proteins containing the hidden markov model PF00765 and PF03472 respectively. Interproscan [15] was used to validate each of the HMM matches. Proteins matching with PF00765 (putative LuxIs) were checked for domains IPR001690 and IPR018311, while proteins matching with PF03472 (putative LuxRs) were checked for domains IPR016032, IPR005143, IPR000792, and IPR036388. These domains are present in nearly all functional LuxI and LuxR proteins respectively. All validated homologs were further scrutinized by aligning them with canonical LuxR and LuxI proteins respectively via ClustalOmega [16]. Certain residues in the alignment were compared against conserved sites identified [17] for further characterization of homology and functionality [18]. To determine the status of LuxR solos in the SD series strains, 10 kbp regions centered around each of the validated luxR homologs were analysed for the presence of *luxI* homologs and visualized by Easyfig [19].

### Results and discussion

The genome sizes of the strains sequenced in this study range between 2.3 to 6.9 megabases with GC content and N_50_ values ranging from 36.10 to 73.22% and 22,000 to 1,041,000 bp, respectively (Table 1). To classify each strain, the 5S, 16S, 23 s rRNA gene sequences were extracted from each genome using BARRNAP (http://www.vicbioinformatics.com/software.barrnap.shtml) and searched against the NCBI database using BLASTN. Species-level identification was also performed using JSpecies [13]. If the output of the BLASTN search corresponded to a species within the JSpecies or NCBI database then the genome in the database was used to calculate the ANI value. The taxonomy information is present in Table 1.

**Table 1:** Genome annotation information for the SD strains

Genbank accession no.	Organism, South Dakota (SD) No.	Genome Size (bps)	No. of contigs	GC %	N_50_ (bp)	Genome Coverage (X)	Based on Pairwise Tetra-correlation test of JSpecies WS	ANI^a^	Quorum-sensing players *luxI*^a^ LuxR^b^	
JADDRN000000000	*SD018*	6,255,096	86	66.90	178,584	68.40	*Delftia *sp.* RIT313*	97.81	−	−
JADILJ000000000	*SD072*	3,885,184	122	71.60	69,363	80.53	*Rathayibacter caricis DSM 15,933*	87.98	−	−
JADILK000000000	*SD075*	5,379,599	166	40.20	96,640	71.10	*Bacillus simplex NBRC 15,720* = *DSM 1321 NBRC 15,720*	94.04	−	−
JADILL000000000	*SD083*	6,204,245	111	66.95	99,487	74.01	*Delftia lacustris*	94.45	−	−
JADIJK000000000	*SD088*	5,371,606	81	38.44	166,199	92.75	*Bacillus galactosidilyticus*	80.73	−	−
JADIJL000000000	*SD090*	5,485,233	151	36.10	107,620	68.48	*Pseudogracilibacillus auburnensis*	97.49	−	−
JADIJM000000000	*SD115*	6,977,688	115	65.9	218,129	57.68	*Achromobacter xylosoxidans*	88.47	−	+ 1 solo
JADIJN000000000	*SD129*	4,909,100	54	53.62	448,601	64.75	*Ochrobactrum pseudogrignonense*	84.43	+	+ , + , + 2 solos
JADIJO000000000	*SD226*	2,375,156	209	73.22	21,313	113.46	*Micrococcus luteus (GCA_000828165) 2385*	97.48	-	-
JADIJP000000000	*SD274*	5,158,613	99	66.1	145,262	61.48	*Methylobacterium *sp.* Leaf86*	97.15	-	+ 1 solo
JADIJQ000000000	*SD287*	3,022,529	66	68.57	39,964	143.85	*Tessaracoccus flavescens*	71.13	-	-
JADIJR000000000	*SD291*	3,528,957	88	69.24	119,791	92.89	*Microbacterium oxydans*	80.59	-	-
JADIJS000000000	*SD316*	4,833,691	24	53.52	1,040,937	59.06	*Ochrobactrum pituitosum CCUG 50,899*	96.53	-	+ , + , + 3 solos
JADIJT000000000	*SD340*	6,349,462	387	64.10	40,898	47.08	*Acidovorax *sp.* root275*	87.94	+ , +	+ , +

ANI represents the average nucleotide identity calculated by JSpecies. Dashes indicate that the gene for comparison was not sequenced in the corresponding type strain

^a^The antiSMASH platform [12] was used to identify *luxI* gene(s) in each genome

^b^The GenBank accession number AFP89744.1 was used to identify *luxR*(s) gene(s)

**Fig. 1:**
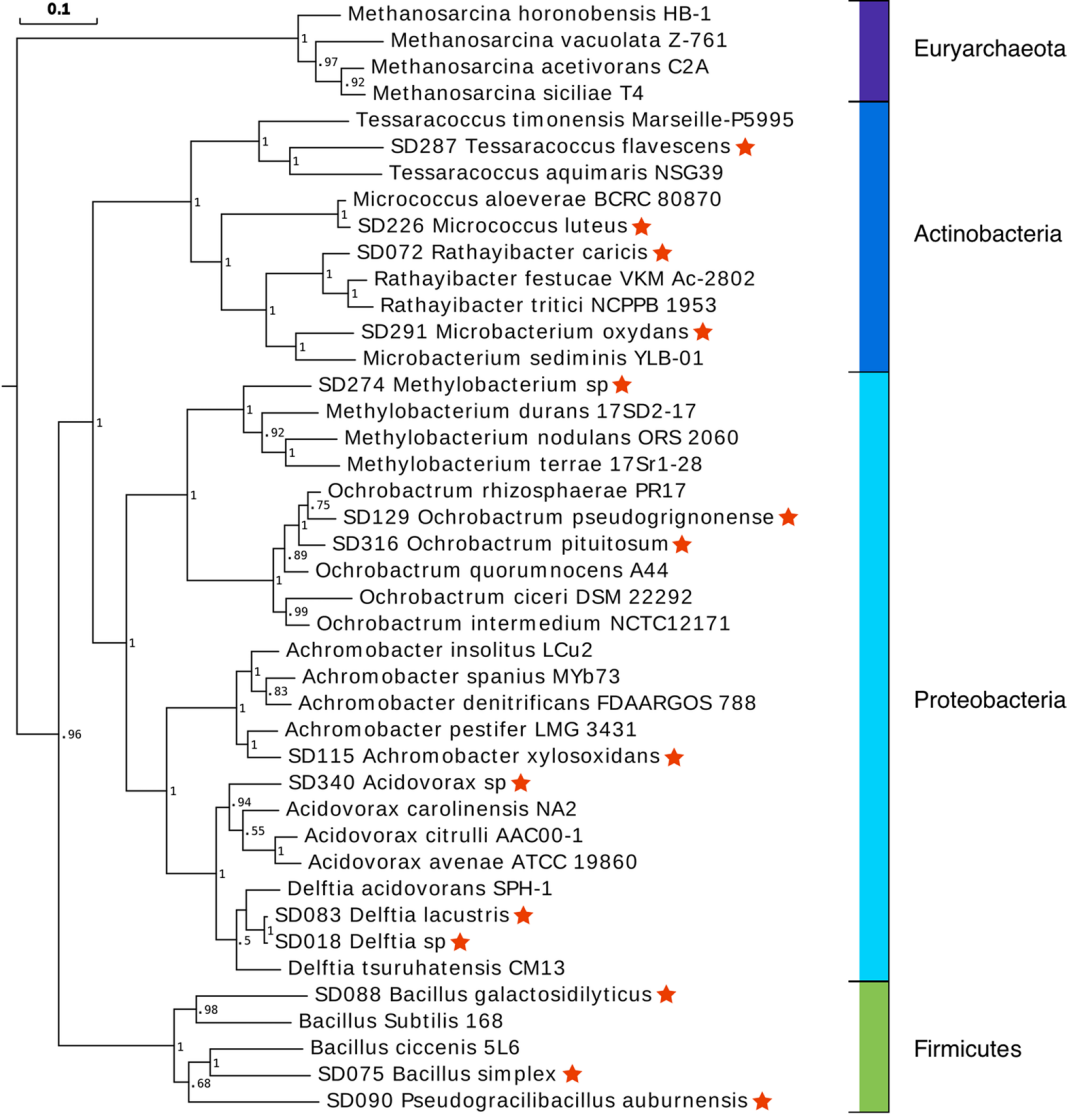
Evolutionary placement of fourteen WCNP strains. The fourteen strains sequenced in this study are denoted by a red star next to the strain name. Phylogeny was based on PhyloPhlAN analysis of four hundred of conserved domains scattered throughout the genome sequences. These strains showed phylogenetic diversity, spanning Proteobacteria, Actinobacteria, and Firmicutes. An archaebacterial clade, Methanosarcina, was selected as an outgroup

ANI analysis and JSpecies package [13] were used to investigate the species circumscriptions of the fourteen SD strains (Table 1). An ANI value in the range of 95% to 96% is the accepted cut-off threshold for species-species delineation [13]. Only five of the fourteen genomes produced an ANI value at > 96%, those being strains SD018, SD090, SD226, SD274, SD316. Strains SD075 and SD083 had ANI values approximately 94 to 95%, setting these two strains in the *transitionary* zone [13]. The remaining seven SD strains (072, 088, 115, 129, 287, 291, and 340) produced ANI values spanning 71% to 89% within the *different* species zone (uncertain taxonomic status) putatively indicating that these strains could represent new species (Table 1). Ten monophyletic groups encompass the fourteen WCNP strains, of which three are located in the Firmicutes, four in the Actinobacteria and seven in the Proteobacteria phyla (Fig. 1). This genomic information warrants further re-classification investigations.

Strain SD340, an *Acidovorax* species, was found to have an abnormality in one of its canonical LuxI/R QS systems. This abnormality is with regards to *luxI* homolog localized on contig 4, which was initially discounted due to the missing autoinducer synthesis conserved site, IPR018311. Further analysis indicated, however, that this LuxI could still be a functional autoinducer synthase. PFAM analysis identified the protein as being in the "autoinducer synthase family", achieving a bit score of 81.3 with e-value 6.0e-23. Furthermore, this protein, when aligned with the canonical LuxI proteins, demonstrated complete consensus with the conserved residues as described in Fuqua and Greenberg [20]. Further evidence provided by Lim et al. [21] confirmed the existence of functional LuxI proteins lacking the IPR018311 domain. Due to complete consensus of the conserved residues and validation in clinical isolate *Pandoraea pnomenusa* RB38 of the ppnI [21], we propose that the *luxI* on contig 4 of *Acidovorax* sp. strain SD340 is an authentic AHL synthase gene (Additional file 1). Further investigations into the AHL synthase activity encoded by this *luxI* are currently underway.

**Fig. 2:**
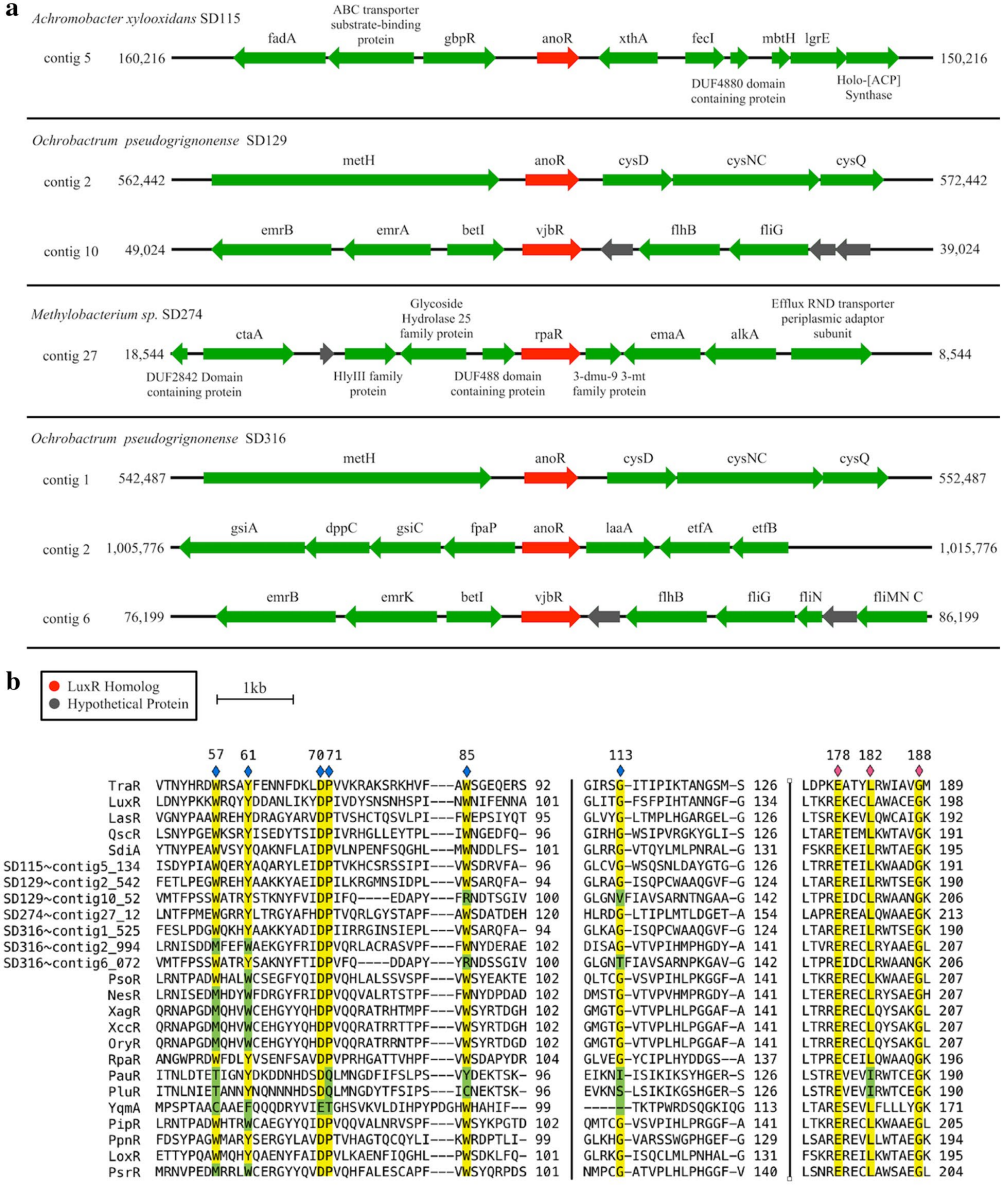

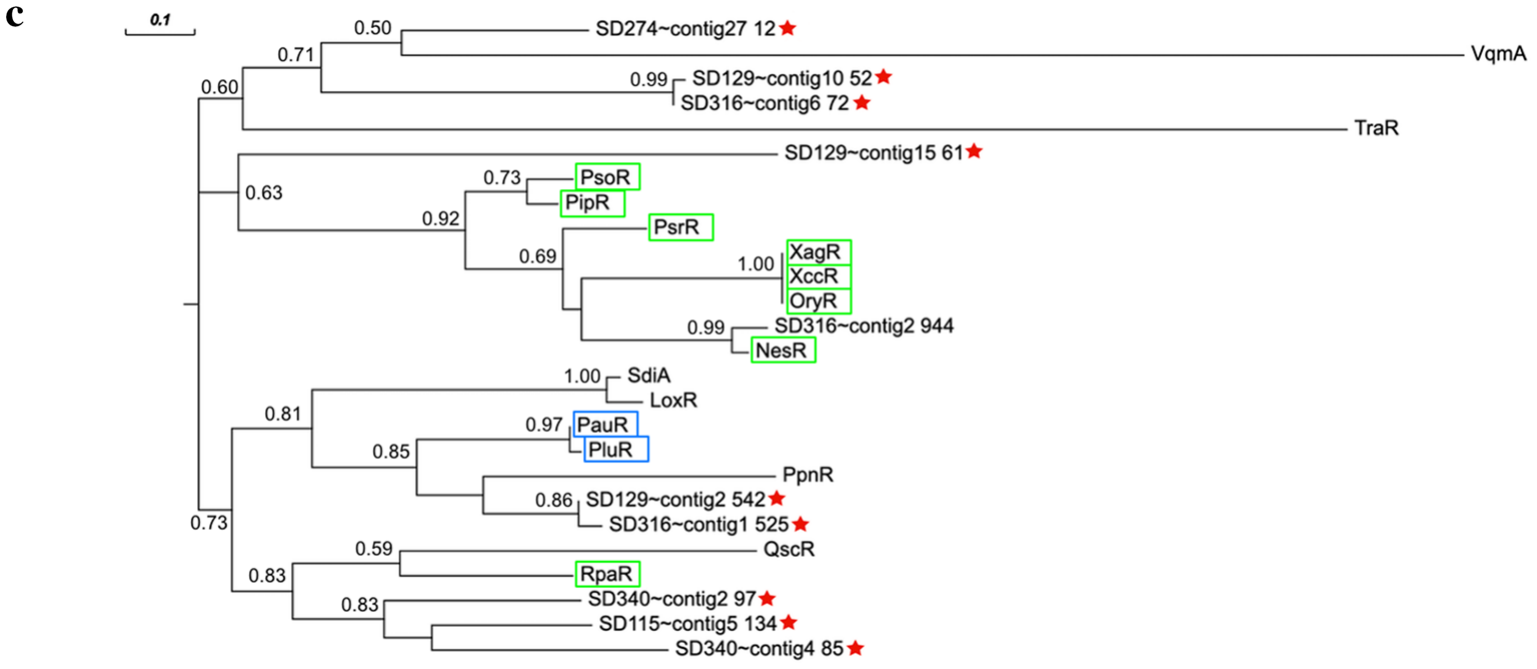
Detection and analysis of LuxR solos. **a** 10 kbp genetic region surrounding identified luxR homologs (luxR solos) (red) having no corresponding *luxI* homologs. **b** Alignment of putative LuxR homologs with canonical LuxR homologs using clustalOmega. Highlighted residues correspond to invariant sites in authentic LuxR-type AHL-mediated transcriptional regulators [21]. Residues highlighted in yellow match with the consensus, whereas those highlighted in green vary from the consensus. Regions marked with a blue diamond are involved in ligand-binding. Regions marked with a pink diamond are involved in DNA-binding. Residues are numbered based on the sequence of TraR. **c** Evolutionary placement of LuxR family transcriptional regulators. LuxR homologs from the WCNP strains are denoted by a red star. LuxR homologs surrounded by a green line box are LuxR solos from plant-associated bacteria (PAB). LuxR homologs surrounded by a blue line box are neither AHL-like or PAB

A total of seven luxR solos have been identified in SD115, SD129, SD274 and SD316 and their gene neighborhoods are shown (Fig. 2a, Additional file 2). The three conserved residues of the DNA-binding domains E178, L182 and G188 are conserved in all seven SD strain LuxR solo homologs (Fig. 2b). Alignment of the identified LuxR solo homologs from SD strains shows substitution in the LuxR homolog (vjbR) from SD316 (contig 2_994) in the highly conserved amino acids in the regulatory domains W57M and Y61W that is similarly reported in PAB LuxR solos (Fig. 2b). PAB LuxR solos e.g., NesR, XagR, OryR, PsoR and others (Fig. 2c) form a robust monophyletic group with LuxR solo of SD316 (contig2_994). The W and M substitutions may be involved in binding to plant-based compounds, as the substitutions are present in OryR and partially present in PsoR, from two PAB known to have an inter-kingdom exchange with plants [22, 23].

All of the seven putative SD strain LuxR solos contain the conserved amino acids D70, P71, and E178, L182 and G188. A LuxR solo identified from SD316, on contig 2_994, has substitutions W85M and Y61W (Fig. 2b), identical to the LuxR solo, PsrR, from the plant endophyte *Kosakonia* sp. PsrR belongs to the PAB subfamily of LuxR solos and was shown to be involved in root endosphere colonization [24]. Furthermore, substitutions were observed in two of the seven LuxR solo homologs from SD129 (contig 10_52) and SD316 (contig 6_72) in which the conserved amino acids in regulatory domains contained substitutions W85R and for G113 residue, V and T, respectively (Fig. 2b). These amino acid substitutions represent novel changes not reported in other LuxR solo proteins and may reflect specificities required for the unknown binding molecule(s) for these two LuxR solo regulatory proteins. Building on this trend, Coutinho and coworkers showed that an ethanolamine derivative from cottonwood tree leaf macerates activates the *Pseudomonas* sp. GM79 *pipA* expression at extremely low concentrations (10 pM) and that the LuxR solo, PipR is required for *pipA* activation [25, 26].

Comparison of the *Ochrobactrum pseudogrignonense* strains SD129 and SD340 with those species in the NCBI database show a staple pattern with three *luxR* genes and one *luxI* gene. The *luxI*, whenever present, appears to always have a proximal *luxR* (Additional file 3).

### Conclusion

We hypothesize that the LuxR solos reported here could potentially be responsive to AHLs or different signals produced by neighboring species or signals in the aquifer water and coordinate regulation of gene expression, thus potentially playing important roles in the ecology and persistence of these species in this pristine aquifer.

## Limitations

This work is from draft genome assembly of bacterial strains.

The possible presence of plasmids in strain cannot be clearly identified.

## Supplementary Information

**Additional file 1.** Detection and analysis of LuxI synthases. (**A)** 10kbp genetic region surrounding identified luxR homologs (red) having corresponding LuxI homologs (blue) in SD129 and SD340. **(B)** Interproscan output of a successfully validated *luxI* homolog. Each accession number corresponds to a detected protein domain. **(C)** Alignment of putative LuxI homologs with canonical LuxI homologs using clustalOmega. Residues highlighted in yellow are invariant sites in validated LuxI-type autoinducer synthases (Fuqua and Greenberg, 2002). Residues are numbered based on the sequence of TraI.

**Additional file 2.** Interproscan output of a successfully validated luxR homolog. Each accession number corresponds to a detected protein domain.

**Additional file 3.** Genomic analyses of eight *Ochrobactrum pseudogrignonense* strains. Analysis of strains available on NCBI and comparison to SD129 and SD316 reveal a commonality in the presence of *luxR* and *luxI* genes^1^.

## Data Availability

The genome sequences of the strains described in this study have been deposited in the GenBank database. The accession numbers and annotation features are presented in Table 1. Direct links are below: SD018—https://www.ncbi.nlm.nih.gov/nuccore/JADDRN000000000 SD072—https://www.ncbi.nlm.nih.gov/nuccore/JADILJ000000000 SD075—https://www.ncbi.nlm.nih.gov/nuccore/JADILK000000000 SD083—https://www.ncbi.nlm.nih.gov/nuccore/JADILL000000000 SD088—https://www.ncbi.nlm.nih.gov/nuccore/JADIJK000000000 SD090—https://www.ncbi.nlm.nih.gov/nuccore/JADIJL000000000 SD115—https://www.ncbi.nlm.nih.gov/nuccore/JADIJM000000000 SD129—https://www.ncbi.nlm.nih.gov/nuccore/JADIJN000000000 SD226—https://www.ncbi.nlm.nih.gov/nuccore/JADIJO000000000 SD274—https://www.ncbi.nlm.nih.gov/nuccore/JADIJP000000000 SD287—https://www.ncbi.nlm.nih.gov/nuccore/JADIJQ000000000 SD291—https://www.ncbi.nlm.nih.gov/nuccore/JADIJR000000000 SD316—https://www.ncbi.nlm.nih.gov/nuccore/JADIJS000000000 SD340—https://www.ncbi.nlm.nih.gov/nuccore/JADIJT000000000.

## Abbreviations

AHLs: Acyl homoserine lactones
AntiSMASH: Antibiotics and secondary metabolite analysis shell
HTH: Helix-turn-helix
PAB: Plant-associated bacteria
WCNP: Wind Cave National Park
