# Supplementary figures and images for: Genomic characterization of bacteria from the ultra-oligotrophic Madison aquifer: insight into the archetypical LuxI/LuxR and identification of novel LuxR solos
Source: BMC Res Notes. 2021 May 8;14:175. doi: 10.1186/s13104-021-05589-6 (PMC8105983; doi:10.1186/s13104-021-05589-6)

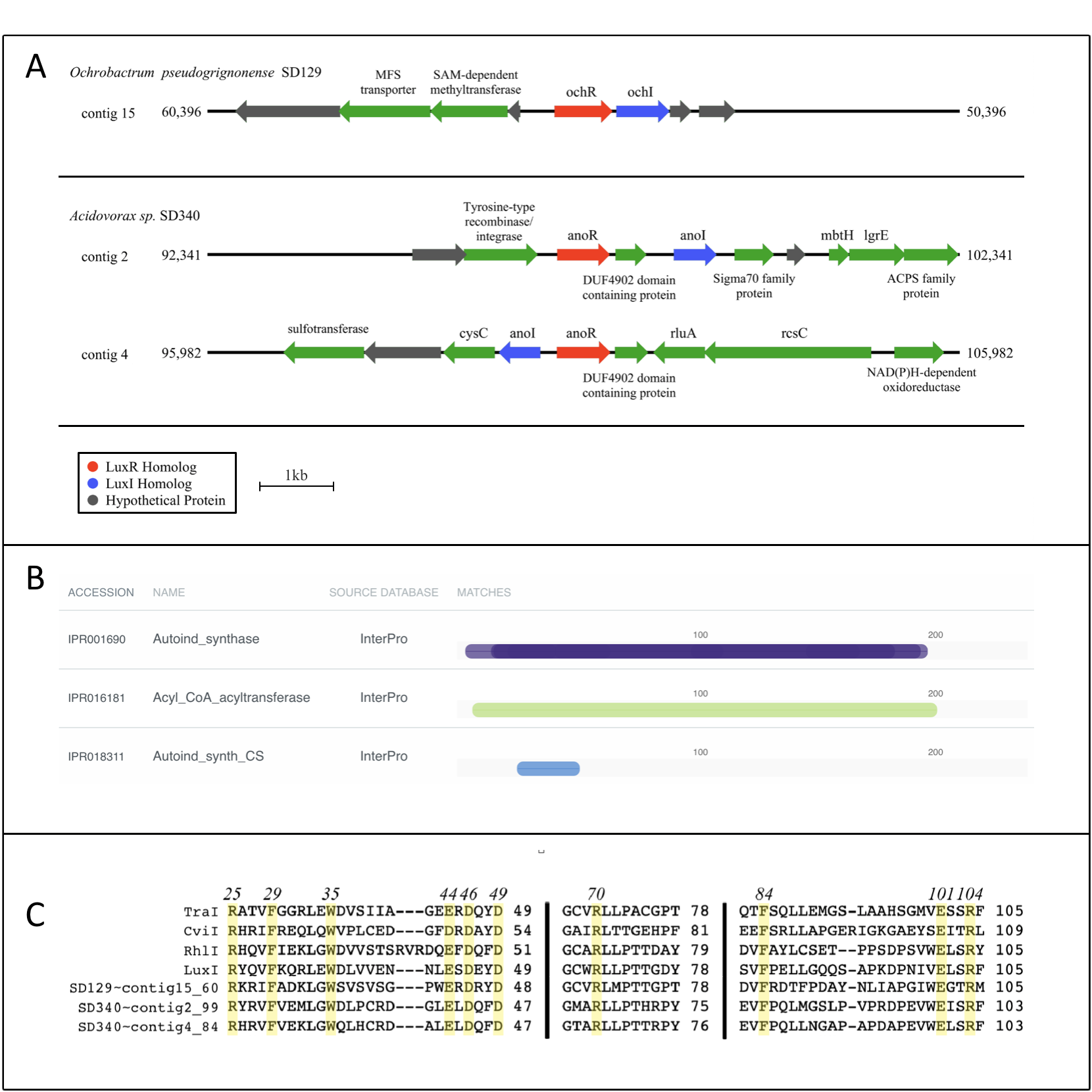

Supplement: Supplementary file 1 — Additional file 1. Detection and analysis of LuxI synthases. (A) 10kbp genetic region surrounding identified luxR homologs (red) having corresponding LuxI homologs (blue) in SD129 and SD340. (B) Interproscan output of a successfully validated luxI homolog. Each accession number corresponds to a detected protein domain. (C) Alignment of putative LuxI homologs with canonical LuxI homologs using clustalOmega. Residues highlighted in yellow are invariant sites in validated LuxI-type autoinducer synthases (Fuqua and Greenberg, 2002). Residues are numbered based on the sequence of TraI. [file 13104_2021_5589_MOESM1_ESM.docx]

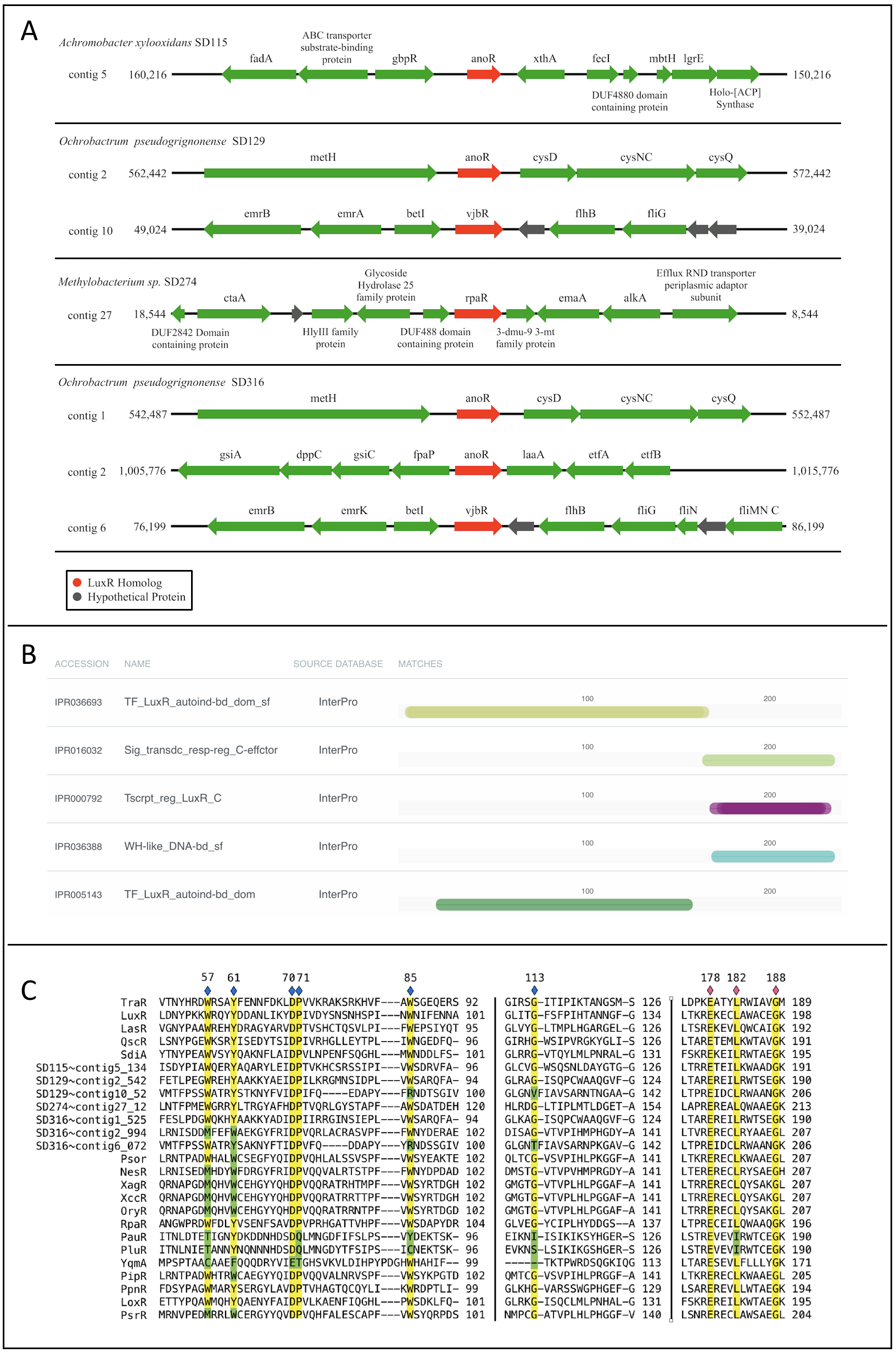

Supplement: Supplementary file 2 — Additional file 2. Interproscan output of a successfully validated luxR homolog. Each accession number corresponds to a detected protein domain. [file 13104_2021_5589_MOESM2_ESM.docx]
