# Supplementary material for: Genomic characterization of bacteria from the ultra-oligotrophic Madison aquifer: insight into the archetypical LuxI/LuxR and identification of novel LuxR solos
Source: BMC Res Notes. 2021 May 8;14:175. doi: 10.1186/s13104-021-05589-6 (PMC8105983; doi:10.1186/s13104-021-05589-6)
Supplement: Supplementary file 3 — Additional file 3. Genomic analyses of eight Ochrobactrum pseudogrignonense strains. Analysis of strains available on NCBI and comparison to SD129 and SD316 reveal a commonality in the presence of luxR and luxI genes1. [file 13104_2021_5589_MOESM3_ESM.docx]

**Additional File 3:** **Table 1.** **Genomic analyses of eight *Ochrobactrum pseudogrignonense* strains.** Analysis of strains available on NCBI and comparison to SD129 and SD316 reveal a commonality in the presence of *luxR* and *luxI* genes**^1^**.

| **Accession #** | **Strain** | **Source** | **Lux pairs** | **LuxR solos** |
| --- | --- | --- | --- | --- |
| JADIJN000000000 | SD129 | Madison aquifer**^2^** | 1 | 2 |
| JADIJS000000000 | SD316 | Madison aquifer**^2^** | 0 | 3 |
| ASM165248 | K8 | Tropical soil | 1 | 2 |
| CCUG30716 | CCUG30717 | Human blood | 1 | 2 |
| ASM297523 | MYb58 | *C. elegans* | 1 | 2 |
| ASM1310922 | SHIN | Human tissue | 1 | 2 |
| CCUG43891 | CCUG43892 | Amniotic fluid | 1 | 2 |
| ASM297929 | MYb37 | *C. elegans* | 1 | 2 |
| ASM297921 | MYb70 | *C. elegans* | 1 | 2 |

**^1^**Genomic analyses of eight *Ochrobactrum pseudogrignonense* strains reveal a commonality in the presence of *luxR* and *luxI* genes. The pattern of three *luxR* genes and one *luxI* gene seems to be a relative staple of the species. The *luxI*, whenever present, seems to always have a companion *luxR* adjacent to it. The *Ochrobactrum* genus is unusual in that it has not one, but two chromosomes. The type strain K8 has had these two chromosomes explicitly identified, and in this strain K8 it has been determined that one chromosome contains two *luxR* solo genes, while the other chromosome contains the *luxI/luxR* pair. This pattern may persist in the other strains. *luxR* and *luxI* genes were identified and validated using the same procedures as was performed for the SD series strains.

**^2^**Strains from this work.
